# A Cell-Based Functional Assay Calibrated for Analysis of *MSH6* and *MSH2* Mismatch Repair Gene Variants

**DOI:** 10.1155/humu/3923193

**Published:** 2025-09-06

**Authors:** Elizabeth Szabo, Emily Blackburn, Olivia N. Amodeo, Samantha Nadeau, Alexander A. Radecki, James P. Grady, Abhijit Rath, Christopher D. Heinen

**Affiliations:** ^1^Center for Molecular Oncology, UConn Health, Farmington, Connecticut, USA; ^2^Department of Molecular and Cell Biology, University of Connecticut, Storrs, Connecticut, USA; ^3^Department of Molecular Biology & Biophysics, UConn Health, Farmington, Connecticut, USA; ^4^Department of Public Health Sciences, UConn Health, Farmington, Connecticut, USA

**Keywords:** CRISPR gene editing, DNA damage response, functional assay, Lynch syndrome, microsatellite instability, mismatch repair, variant of uncertain significance

## Abstract

Variants of uncertain significance (VUS) in the DNA mismatch repair (MMR) genes can confound the diagnosis and treatment of suspected Lynch syndrome (LS) patients. To aid the reclassification of VUS, we developed the in cellulo analysis of MMR variants (inCAMA) and used known control variants to calibrate this assay such that results can be readily applied as functional evidence by expert classification panels. We used CRISPR gene engineering to introduce known pathogenic and benign variants into the *MSH6* or *MSH2* loci in human embryonic stem cells and assessed their effects on cellular MMR repair and damage response functions. Our functional assay successfully discerned known pathogenic and benign variants. Using these results and performing a linear regression analysis with available odds of pathogenicity scores for the known calibration variants, we created equations that can generate a functional odds of pathogenicity score for any future *MSH6* or *MSH2* variant tested. In summary, inCAMA represents a new, calibrated assay for testing the function of virtually any *MSH6* or *MSH2* variant. The conversion of assay results directly into odds of pathogenicity scores makes it possible to use any PS3 or BS3 evidence strength level toward the reclassification of VUS.

## 1. Introduction

Lynch syndrome (LS) (MIM# 120435) is an autosomal dominant condition that increases patients' risk for cancer, predominantly colorectal cancer (CRC) and endometrial cancer [1]. LS patients make up about 2%–4% and 1%–5% of all CRC and endometrial cancer cases, respectively [2–5]. Patients may be considered for LS diagnosis due to early onset of cancer, significant family history of CRC and endometrial cancer, and certain tumor characteristics. Diagnosis is confirmed by performing genetic testing to identify a germline pathogenic variant in one of the four major DNA mismatch repair (MMR) genes, *MLH1* (MIM# 120436), *MSH2* (MIM# 609309), *MSH6* (MIM# 600678), and *PMS2* (MIM# 600259). However, the pathogenicity of some identified variants is unclear, leading to difficulty with diagnosis and, therefore, clinical management for patients and their relatives. These variants, the vast majority being missense variants, are referred to as variants of uncertain significance (VUS).

To guide the assessment of pathogenicity for an identified MMR gene patient variant, an evaluative framework had been proposed by the International Society for Gastrointestinal Hereditary Tumors (InSiGHT) Variant Interpretation Committee (VIC) [6] that was built off of the American College of Medical Genetics and Genomics (ACMG) and the Association for Molecular Pathology (AMP) guidelines for variant classification [7]. These guidelines describe the various pieces of evidence that can be used, including segregation of the variant in affected family members, frequency of the variant in the population, tumor pathology features, and in silico predictions. A qualitative descriptor is then assigned to each piece of evidence based on the strength of that evidence. Combining different pieces of evidence, as instructed by the guidelines, leads to an overall classification of pathogenic, likely pathogenic, VUS, likely benign, or benign.

In addition to clinical and family history data, another piece of evidence the guidelines consider is the result of laboratory-based functional assays in which the direct effect of the variant on MMR function is assessed. The MMR pathway is responsible for identifying and rectifying misincorporated nucleotides in the daughter strand during DNA replication, thus contributing to the overall stability of the genome [8–10]. MMR is also known to repair insertion/deletion loops within simple nucleotide repeat regions, known as microsatellites. This repair mechanism prevents potential frameshift mutations of microsatellite sequences in the coding region of cancer-driving genes [11–14]. Additionally, MMR initiates cell cycle arrest and/or apoptotic signaling in response to persistent DNA damage, such as methylation of guanines caused by S_N_1 DNA alkylating agents [15–17].

Numerous laboratory approaches have been used for testing the impact of MMR gene variants on function, including the use of model systems like yeast and mice, in vitro experiments in the test tube, and studies in human cells [18–20]. Recently, the InSiGHT VIC, now functioning as a ClinGen Expert Panel, released new guidelines for the classification of MMR gene variants, which describe how to utilize historical functional data for classification purposes. However, for prospective functional testing of variants, they now recommend utilizing carefully calibrated assays using a sufficient number of known pathogenic and benign controls [21]. The results of these assays can be directly converted into a probability or odds of pathogenicity (OddsPath_Functional) score [22, 23]. Thresholds provided in Brnich et al. describe how to convert the OddsPath_Functional scores into corresponding descriptive evidence: supporting, moderate, or strong evidence of pathogenicity (PS3) and moderate or strong evidence for benign status (BS3) for use with the new ClinGen/InSiGHT guidelines [24]. To date, there is one calibrated assay that is capable of reaching these evidence categories for variants in the MMR genes. The cell-free in vitro MMR assay (CIMRA) involves the in vitro transcription and translation of variant-containing cDNA, which is then mixed with human nuclear extracts from MMR-deficient cell lines and an artificial DNA template with an engineered G/T mismatch [22, 25]. Though carefully calibrated and likely suitable for testing many missense variants, the artificial nature of this assay does not fully recapitulate all aspects of MMR function. Furthermore, this assay is unsuitable for studying noncoding variants and other variants that may impact RNA expression or splicing and variants that impact aspects of MMR protein function such as stability, localization, and chromatin interaction.

To address these concerns, we have developed an assay where the effects of variants can be tested in a cellular environment. The in cellulo analysis of MMR variants (inCAMA) uses clustered regularly interspaced short palindromic repeat (CRISPR) genome editing to engineer the variant directly into the endogenous MMR gene loci [26, 27] to assess their effects on MMR repair and damage response functions. This cellular approach allows us to assess the impact of virtually any MMR gene variant on any aspect of MMR RNA and protein function. inCAMA joins another previously published assay that tests MMR variant function within cells utilizing the endogenous gene loci [28–30]. However, this prior approach utilized mouse embryonic stem cells to examine only a small number of variants to date and has not been calibrated for extensive use in variant classification. By contrast, we have recently calibrated the human cell-based inCAMA for use with variants in the *MLH1* MMR gene [27] making it the second MMR functional assay capable of generating all the PS3 and BS3 evidence levels for *MLH1*. Since one cannot assume in a multigenic condition such as LS that calibrating an assay for one gene is equivalent to calibrating it for the other genes implicated, we have now sought to calibrate inCAMA for use with variants in the *MSH6* and *MSH2* MMR genes. In this study, we used a subset of known control variants for each gene to calibrate our assay and then utilized it to generate OddsPath_Functional scores for a second set of variants. By establishing a statistically tested cell-based functional assay, a wider variety of MMR gene variants can now be assessed with this approach to aid their classification by the ClinGen/InSiGHT Expert Panel.

## 2. Methods

### 2.1. Generation of *MMR* Variant-Expressing Cell Lines

H1 human embryonic stem cells (hESCs) were obtained from the University of Connecticut Cell and Genome Engineering Core. Cells were cultured as previously described [26] except cells were cultured in mTeSR+ stem cell media (STEMCELL Technologies), which was replenished every 2 days. For each *MSH6* (NCBI Gene ID: 2956, RefSeq: NM_000179) or *MSH2* (NCBI Gene ID: 4436, RefSeq: NM_000251.3) variant created for this study, a single guide RNA (sgRNA) near a protospacer adjacent motif (PAM) was designed to target the desired genomic change, and its efficiency was predicted using the in silico program CRISPOR [31] (Supporting Information 2: Tables S1 and S2). The targeting approach was described previously [26] with the following modifications: following transfection, cells were plated as single cells and treated with homologous direct repair enhancer media, containing ROCK inhibitor (Y-27632, Selleck Chemicals), L755507, SCR7, and HDR Enhancer V2 (IDT), for 24 h. Surviving cells were treated with CloneR (STEMCELL Technologies) as recommended for the next 24 h. Colonies grown from single cells were picked after approximately 7 days, and their genotype was confirmed by Sanger sequencing using primers described in Supporting Information 2: Tables S3 and S4.

The top five most likely off-target binding sites for each sgRNA were determined using the CRISPOR tool [31]. Each off-target site was PCR amplified and analyzed by Sanger sequencing in both the variant cell line and the parental wild-type (WT) H1 cells from which it was generated to confirm no off-target aberrations caused by the CRISPR editing.

### 2.2. Immunoblotting

Approximately 1 million cells were lysed in 120 *μ*L RIPA buffer in the presence of protease inhibitors. Thirty micrograms of protein was run on an 8% SDS-PAGE gel and probed with anti-MSH6 (Bethyl #A300-023A, 1:1000), anti-MSH2 (BD Pharmingen #556349, 1:1000), and anti-Actin (Santa Cruz Biotechnology #sc-8432, 1:1500) antibodies. Quantification of MSH6 and MSH2 levels in each cell line was compared to MSH6 and MSH2 levels in WT hESCs after being normalized to the actin protein loading control via ImageJ software.

### 2.3. Exon Inclusion Assay

To examine the effects of variants on RNA splicing, exon inclusion analyses were performed as described previously [27]. The indicated cell lines were treated with 20 *μ*g/mL cycloheximide (CHX) (Sigma-Aldrich) for 4 h to accumulate potentially unstable transcripts. Cells treated with CHX were collected, and RNA extraction was performed using a Quick-RNA Microprep Kit (Zymogen Research). Five hundred nanograms of total RNA was used in a reverse transcription reaction using the iScript cDNA synthesis kit (Bio-Rad). For this study, the variant-containing Exon 9 was PCR amplified using these primers in the flanking exons: **Forward**: TTGTTGAATTAAGTGAAACTGC (Exon 7) and **Reverse**: ATGGACAGCTTCAGCATCTAC (Exon 10). The PCR products were analyzed on a 1% agarose gel.

### 2.4. Cell Survival Assay

Approximately 3,000 cells were plated in individual wells of a Matrigel-coated 96-well plate along with 5 *μ*M ROCK inhibitor. The media was replaced without ROCK inhibitor the next day, and the cells were allowed to grow overnight. Cells were initially treated with 25 *μ*M O^6^-benzylguanine (O^6^-BG, Sigma) for 1 h to inhibit the action of the O^6^-methylguanine-DNA methyltransferase and then treated with 1 *μ*M of the S_N_1 DNA alkylating agent *N*-methyl-*N*⁣′-nitro-*N*-nitrosoguanidine (MNNG, obtained from the National Cancer Institute Chemical Carcinogen Reference Standard Repository) for 48 h in the presence of O^6^-BG. Survival was assessed using the 3-(4,5-dimethylthiazol-2-yl)-2,5-diphenyltetrazolium bromide (MTT) assay (Thermo Fisher Scientific) following the manufacturer's instructions. Assays were performed in technical triplicates and repeated at least three times.

### 2.5. Microsatellite Instability (MSI) Analysis

Cells were grown for approximately 19–21 passages and then diluted to isolate single-cell colonies. Following 7 additional days in culture, ~32 colonies were picked for each cell line and genomic DNA was extracted. PCR primer pairs for six mononucleotide loci as indicated below were designed with either 6-FAM or Hex-labeled fluorescent forward primers (Supporting Information 2: Table S5). For *MSH2* variant lines, a multiplex reaction (NR-27 and BAT-25) and a single reaction (BAT-26) were performed on all clones. For *MSH6* variant lines, three single reactions (BAT-40, MONO-27, and MONO-51) were performed. PCR products for NR-27, BAT-25, and BAT-26 reactions were combined for fragment analysis by capillary electrophoresis (Genewiz). PCR products for BAT-40 and MONO-27 reactions were similarly combined, while MONO-51 reactions were examined individually. The software program Geneious Prime was used to identify instability for each marker via manual scanning of individual peaks by at least two blinded reviewers.

### 2.6. Statistical Analyses

The assay results collected were used for hierarchical clustering as we have described previously [27], specifying a two-cluster solution in the CLUSTER procedure in SAS 9.4 (SAS Institute Inc.) All *MSH6* variants were analyzed simultaneously, and the two best clusters were identified. This was done similarly for *MSH2* variants. Clusters were created independently for percentage survival scores for the MNNG survival assay and the fraction of unstable clones for each MSI marker. We then converted our functional results into odds of pathogenicity (OddsPath) functional scores (OddsPath_Functional) as we have described previously [27]. Briefly, we first obtained previously calculated OddsPath_Nonfunctional scores for each *MSH6* or *MSH2* calibration variant from the InSiGHT MMR Integrative Evaluation Tool (http://insight-database.org/classifications/mmr_integrative_eval.html, Supporting Information 2: Tables S6 and S7). Any prior functional data was excluded from these scores to avoid circularity. Ordinary least squares regression was used to estimate a linear relationship on the log10 scale using the log10 of the previously calculated OddsPath scores as the dependent variable and the results from either the MNNG survival assay or the average MSI result of the three markers as the independent variable.

The equation for the MNNG assay for *MSH2* is

 $$\begin{array}{c} \log10\;\left(\text{OddsPath}\right)=-7.2554+0.1224\left(\text{avg}.\text{survival}\%\right). \end{array}$$

The equation for the MNNG assay for *MSH6* is

 $$\begin{array}{c} \log10\left(\text{OddsPath}\right)=-4.8529+0.0990\left(\text{avg}.\text{survival}\%\right). \end{array}$$

The equation for the MSI assay for *MSH2* is

 $$\begin{array}{c} \log10\left(\text{OddsPath}\right)=-5.9599+11.6173\left(\text{avg}.\text{MSI}\%\right). \end{array}$$

The equation for the MSI assay for *MSH6* is

 $$\begin{array}{c} \log10\left(\text{OddsPath}\right)=-3.6966+8.9268\left(\text{avg}.\text{MSI}\%\right). \end{array}$$

Using these equations and the assay results for each additional variant in our testing set, log10(OddsPath_Functional) scores were calculated. The GENMOD procedure in SAS was used to estimate the linear model parameter estimates and their standard errors, and the predicted log10 (OddsPath_Functional) scores were generated using the PSL procedure. A final OddsPath_Functional score was created by combining the MNNG survival assay score and MSI assay score using a meta-analytic approach with the GENMOD procedure in SAS, which gives a variance-weighted estimate with 95% confidence limits [32].

## 3. Results

### 3.1. Generating *MSH6* and *MSH2* Variants

**Figure 1:**
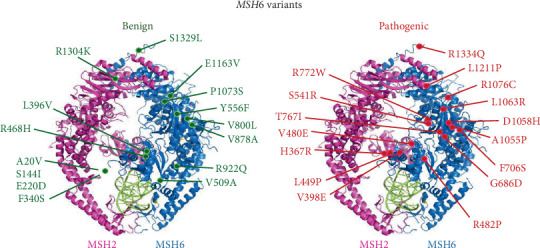
MSH6 variants under study. Representative image of the benign/likely benign and pathogenic/likely pathogenic MSH6 variants mapped to the crystal structure of the human MSH2–MSH6 heterodimer complex (Protein Data Bank ID Code 2O8C).

We identified known benign and pathogenic missense variants for *MSH6* from the Leiden Open Variant Database and InSiGHT MMR Integrative Evaluation tool. These variants were classified by the InSiGHT VIC based on their calculated posterior probability of pathogenicity scores [6, 23]. We found only four benign missense variants and nine pathogenic missense variants originally listed in the database. Due to the low number of known pathogenic and benign variants, we chose to include variants classified as likely pathogenic and likely benign. With this addition, we created a list of six pathogenic/likely pathogenic (P/LP) and six benign/likely benign (B/LB) variants to be used as calibration variants (Figure 1 and Supporting Information 2: Table S6). The variants that were chosen for calibration variants were those that were classified without functional evidence to avoid bias with functional data. We then chose an additional set of 10 P/LP and 9 B/LB variants originally classified similarly to be used as a testing set of variants (Figure 1 and Supporting Information 2: Table S6).

Using CRISPR gene editing in WT H1 hESCs as described previously [27], we generated cell lines for all 31 *MSH6* missense variants. Depending on the availability of different PAM sites near the target nucleotide, we used either Cas9 or Cas12a nucleases, which improved our ability to efficiently generate a greater number of variants. sgRNAs were generated for each variant that were determined to have a low chance of off-target effects by in silico prediction analysis [31]. The presence of the single nucleotide change was confirmed by Sanger sequencing for each variant clone (Supporting Information 1: Figure S1). We also created an *MSH6* knockout (KO) cell line by targeting Exon 4 and allowing for random nonhomologous end joining repair. This led to a cell line in which the MSH6 protein was lost (Supporting Information 1: Figure S2). Additionally, the Top 5 predicted off-target sites for each sgRNA were PCR amplified and Sanger sequenced, revealing no off-target events (Supporting Information 2: Table S8). One *MSH6* variant Gly686Asp required two successive targeting events with two different sgRNAs to successfully generate the desired nucleotide change due to a persistent microhomology-mediated end joining repair of the initial CRISPR-generated double-strand break. For this variant, off-target sites for both sgRNAs were sequenced, and no off-target events were found.

### 3.2. Impact of Variant on MSH6 and MSH2 Protein Levels and mRNA Splicing

To examine for immediate effects of a variant on MSH6 protein expression and/or stability, we performed a Western blot of lysates from all *MSH6* missense variant lines with an anti-MSH6 antibody. As expected, all the *MSH6* B/LB variant cell lines showed levels of MSH6 and MSH2 comparable to the WT levels (Supporting Information 1: Figure S2A,C). The majority of P/LP variants resulted in reduced levels of MSH6 to varying degrees. Notably, Arg482Pro, Gly686Asp, Thr767Ile, Asp1058His, Arg1076Cys, Leu1211Pro, and Arg1334Gln showed significantly less MSH6, suggesting that these variants impact the expression and/or stability of the MSH6 protein (Supporting Information 1: Figure S2B,D).

As MSH6 and MSH2 form a heterodimer and loss of MSH6 protein has been shown to result in reduced MSH2 levels as well [33], we also used an anti-MSH2 antibody to check for MSH2 protein levels in our variant cell lines. Unsurprisingly, we saw that variants that showed reduced levels of MSH6 also showed reduced levels of MSH2. Variant cell lines that had almost no detectable MSH6 protein, the *MSH6* KO, Arg482Pro, and Arg1334Gln lines, showed a reduction of MSH2 levels to ~10%–20% of WT. These results suggest that MSH2–MSH6 protein levels can be impacted by MSH6 missense variants. However, only a few pathogenic variants result in complete loss of MSH6 protein, which is important to consider when evaluating immunohistochemistry results from patient tumors, often a first-line test for MMR deficiency.

**Figure 2:**
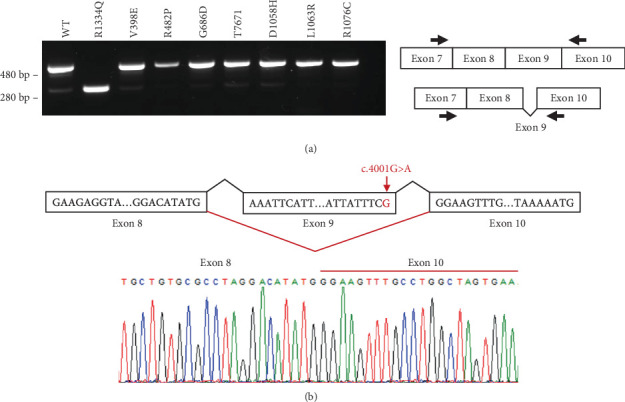
Examination of Exon 9 skipping in variant-expressing cell clones. (a) Reverse-transcribed total RNA from wild-type (WT) and eight different CRISPR-edited cell clones was PCR amplified with primers placed in Exons 7 and 10 of *MSH6* and run on an agarose gel to detect the presence of an alternative splice product as observed in the R1334Q cell line. (b) Sanger sequencing chromatogram displaying the complete skipping of Exon 9 in RNA from R1334Q cells. The nucleotide variant is shown in red. The red lines indicate variant-induced Exon 9 exclusion.

Investigating protein levels through Western blotting only indicates the amount of stable protein present in the cell but does not provide the mechanism by which this reduction occurs. In addition to impacting protein stability, these single nucleotide variants might have impacts on mRNA splicing. As inCAMA involves the genetic modification of the endogenous loci of the *MSH6* gene in human cells, we can also assess the impact of the variant at the mRNA level. To demonstrate this point, we tested the mRNA from Arg1334Gln cells due to the dramatic loss of MSH6 protein in these cells. This single nucleotide change (c.4001G>A) in Exon 9 of *MSH6* has been shown previously to affect splicing [34]. To test its effect in our cell line, we harvested RNA, converted it into cDNA, and then amplified a region between Exons 7 and 10 and compared it to cDNA from WT cells. As seen in Figure 2a, we observed a truncated PCR product in the Arg1334Gln cells compared to the full-length WT product when run on an agarose gel. Sanger sequencing of this PCR product confirmed a complete skipping of Exon 9 in these cells (Figure 2b). Notably, there was a faint band of the same size in the WT sample as well as all other variant cell lines that were tested as additional clonal controls (Figure 2a), suggesting that this skipping of Exon 9 is a naturally occurring alternative isoform; however, the c.4001G>A variant appears to enhance its production.

### 3.3. Variant Impact on DNA Damage Response

**Figure 3:**
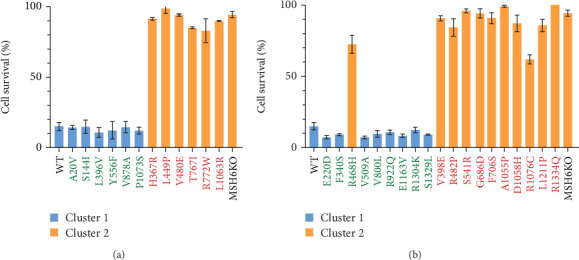
The mismatch repair–dependent DNA damage response in variant cell lines. Cell survival (percentage) for each *MSH6* (a) calibration and (b) testing set variant along with wild-type (WT) and *MSH6* knockout (KO) controls after 48-h treatment with 1 *μ*M of MNNG. The values are represented as the mean ± standard error of the mean; *n* = 3–5. A statistical clustering analysis was performed, grouping the variants into two populations based on their percent survival (blue and orange). Benign/likely benign variants listed in green, pathogenic/likely pathogenic variants listed in red.

While the effects of an *MSH6* variant on protein levels provide some hints as to whether it impacts MMR function, at the core of inCAMA is a direct examination of cellular MMR functions. One repeatedly observed MMR function is the ability of this pathway to result in cell growth arrest and/or cell death in response to certain forms of DNA damage [15–17]. We have shown that hESCs treated with the S_N_1 alkylating agent MNNG undergo rapid apoptosis in an MMR-dependent manner [26, 35]. As such, we have assessed the ability of our control *MSH6* variant cell lines to induce this apoptotic response when treated with 1 *μ*M MNNG for 48 h. As expected, WT cells and all the B/LB *MSH6* calibration variant cells showed extensive cell death in response to MNNG, while MSH6 KO and all P/LP *MSH6* variant cells demonstrated much greater survival (Figure 3). We next performed the same survival assay for the testing set of variant cell lines and similarly saw a distribution of some cell lines that mainly survived and others that showed extensive cell killing (Figure 3). We analyzed the data for all the cell lines in a blinded fashion using a statistical clustering analysis, which groups samples together based on similarity to determine those validation variants that behaved like B/LB or P/LP calibration controls. Indeed, all testing set variant cell lines functioned as expected based on their classification apart from one outlier, Arg468His, which showed considerable survival (72%) despite being an LB variant. To account for possible clonal effects that may occur with CRISPR editing, we re-engineered this variant in a separate CRISPR editing experiment and chose two additional clones (c2 and c3) which also showed increased survival (Supporting Information 1: Figure S3), confirming the apparent loss of the MMR-dependent damage response caused by this variant and the reproducibility of our assay. Although we observed this one discordant result in our testing set, the overall results indicate that measuring cell survival after treatment with MNNG appears to be an informative assay in which the functional results of known B/LB and P/LP variants can be clearly distinguished.

### 3.4. MSI in *MSH6* Variant Cell Lines

To strengthen the ability of inCAMA to distinguish between B/LB and P/LP variants, we also assessed the effect of variants on the hallmark MMR function of repair of slippage events at simple repeat or microsatellite sequences. MSI is a known consequence of MMR-deficient cells and is commonly used to identify tumors associated with LS [36]. We were able to utilize this known function of MMR in our cell model by growing our variant cell lines for 19–21 passages, isolating ~32 single-cell clones and then amplifying gDNA at three different mononucleotide MSI markers.

While the presence of MSI in MSH6-defective tumors has been reported to be reduced when examining the MSI markers traditionally used during clinical testing, markers with longer mononucleotide repeats may be more commonly affected in these tumors [37, 38]. We therefore chose three longer mononucleotide repeats to test the *MSH6* variants: BAT-40, MONO-27, and MONO-51. After determining the percentage of unstable clones in each cell line, we used a similar statistical clustering approach as described above to demonstrate that these three MSI markers can effectively distinguish between B/LB and P/LP variants (Supporting Information 1: Figure S4). While some of the *MSH6* B/LB calibration variants showed low MSI at some markers, the value never exceeded 15% of clones (Table 1). The *MSH6* P/LP calibration variants, meanwhile, showed significant instability in all three markers (Table 1). Likewise, *MSH6* test set variants were also distinguishable, with B/LB variants showing little to no instability and P/LP variants showing high instability (Table 1 and Supporting Information 1: Figure S4). Interestingly, the LB variant Arg468His line that showed unexpectedly high survival following MNNG treatment had low MSI consistent with normal repair function. These results demonstrate that examining MSI can distinguish the function of B/LB and P/LP variants of *MSH6*.

### 3.5. Calibration of inCAMA for *MSH2* Variants

**Figure 4:**
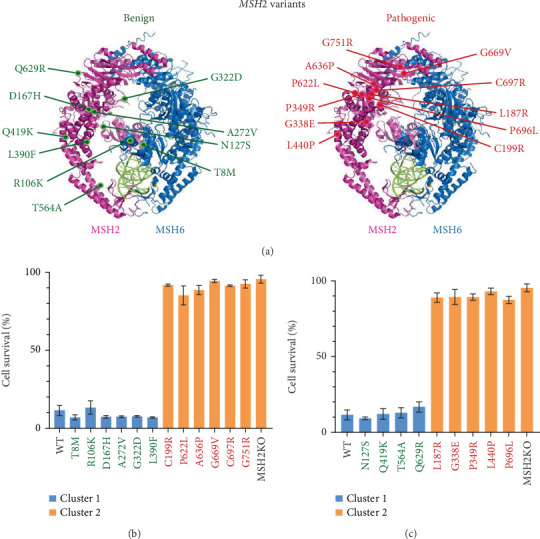
MSH2 variants under study and their DNA damage response. (a) Representative image of the benign/likely benign and pathogenic/likely pathogenic MSH2 variants mapped to the crystal structure of the human MSH2–MSH6 heterodimer complex (Protein Data Bank ID Code 2O8C). Cell survival (percentage) of *MSH2* (b) calibration and (c) testing set variants along with wild-type (WT) and *MSH2* knockout (KO) controls when treated for 48 h with 1 *μ*M of MNNG. The values are represented as the mean ± standard error of the mean; *n* = 3–5. A statistical clustering analysis was performed grouping the variants into two populations based on their percent survival (blue and orange). Benign/likely benign variants listed in green, pathogenic/likely pathogenic variants listed in red.

We previously tested a CRISPR-based approach to examine missense variants in *MSH2* [26]; however, we had not performed the complete analysis as done for *MLH1* [27] and as we have done with *MSH6* in this present study, nor had we calibrated the assays for *MSH2*. Therefore, we identified known B/LB and P/LP missense variants for *MSH2* to be used for calibration and testing (Figure 4a and Supporting Information 2: Table S7).

As we had previously created some of these *MSH2* control variant lines for our initial study [26], we re-examined those cell lines and then generated 11 additional *MSH2* variant cell lines in this study. Using CRISPR gene editing, we introduced these missense variants into our H1 hESCs, similar to the *MSH6* variants. We successfully created these lines, as confirmed by Sanger sequencing (Supporting Information 1: Figure S5) and determined there were no off-target events by sequencing the Top 5 predicted off-target sites (Supporting Information 2: Table S9). We examined these cell lines in the DNA damage response and MSI assays, which are the two components of inCAMA. The cellular response of *MSH2* calibration variants to treatment with 1 *μ*M of MNNG for 48 h showed low survival for all B/LB variants and significant survival for P/LP variants, as expected (Figure 4b). Blinded statistical clustering showed that B/LB and P/LP testing set variants also functioned as expected (Figure 4b). We next evaluated three mononucleotide repeat MSI markers, NR-27, BAT-25, and BAT-26, in *MSH2* variant cell lines. The B/LB variants used for calibration showed no instability in any of these three markers, whereas the known P/LP calibration variants showed significant instability in all three (Table 2). Similarly, we found that the B/LB testing set variant cells had little to no instability and P/LP validation variant lines showed instability in all the markers (Supporting Information 1: Figure S6). When statistical clustering analysis was performed, we found all B/LB cell line results clustered and all P/LP cell line results clustered except for one variant. The Leu440Pro cell line clustered unexpectedly with the normal functioning cell lines for the MSI marker BAT-26, although the number of unstable clones for that marker was still higher than any B/LB variant. The MSI results with the other two markers for this cell line clustered with the abnormally functioning cell lines, more consistent with the reduced function these cells showed in the MNNG survival assay. Ultimately, we have now calibrated inCAMA for use with assessing the functional impact of both *MSH6* and *MSH2* variants.

### 3.6. Functional Odds of Pathogenicity Scores

The current ClinGen/InSiGHT MMR gene variant classification guidelines instruct how calibrated functional assays that result in a quantitative OddsPath_Functional score can be used. To generate such scores from our DNA damage response and MSI assays, we first obtained the known OddsPath_Nonfunctional scores available for our calibration variants that were derived from existing clinical and family segregation data (Supporting Information 2: Tables S6 and S7). We then performed a linear regression analysis with the OddsPath_Nonfunctional score as the dependent variable and the assay readout as the independent variable. To generate an OddsPath score for the MSI assay, the results from the three MSI markers were averaged to create a single MSI OddsPath score for each cell line. A separate OddsPath score was then generated for the MNNG survival data, and the two OddsPath scores were combined into one final OddsPath_Functional score (Table 3 and Supporting Information 2: Tables S10 and S11). All of the OddsPath_Functional scores for the B/LB *MSH6* and *MSH2* variants were < 0.05, consistent with strong evidence of benign status (BS3), except for MSH6 Arg468His, which just missed BS3_Supporting evidence (< 0.48, Table 3). Likewise, all P/LP variants for both genes had OddsPath_Functional scores > 18.7, consistent with strong evidence of pathogenicity (PS3, Table 3).

As we were completing our study, the new ClinGen/InSiGHT classification guidelines were released, and a number of variants in our set were reclassified when using these new rules [21]. Though all of our calibration variants remained B/LB or P/LP, a number of testing set variants were downgraded to VUS (Table 3). However, since many of these variants lacked any prior functional evidence, we are now able to add strong evidence for pathogenic or benign status generated from this study for almost all the variants, which should lead to reclassification for most of them. We predict that the following MSH6 variants can now be reclassified from VUS to LB: Phe340Ser, Val800Leu, Arg922Gln, and Ser1329Leu. Glu220Asp should now be reclassified from LB to B. MSH6 VUS predicted to be reclassified to LP include Val398Glu, Arg482Pro, Ser541Arg, Gly686Asp, Phe706Ser, Ala1055Pro, and Asp1058His. Meanwhile, Arg1076Cys, Leu1211Pro, and Arg1334Gln should be reclassified from LP to P. For MSH2, we predict that Leu187Arg, Pro349Arg, Gly338Glu, Leu440Pro, and Pro696Leu can be reclassified from VUS to LP, while Thr564Ala can be reclassified from LB to B (Table 3).

## 4. Discussion

Providers have been faced with the dilemma of how to treat and care for patients and their families that present with a germline VUS in one of the *MMR* genes. These VUS are classified as such due to a lack of sufficient supporting clinical or family evidence to definitively determine their impact. However, the ACMG/AMP guidelines have included functional assays that can predict the impact of these variants, especially those that reflect the true biological environment, as another tool to help classify these variants [7]. To that end, we have developed inCAMA, a novel functional assay in which we have genetically edited the endogenous loci of the MMR genes *MLH1* [27] and, in this study, *MSH2* and *MSH6* in order to reflect the biological impact these variants have on protein function. By creating this cell-based assay, we can examine repair of endogenous microsatellite regions, as well as the cell response to DNA damage, known functions of the MMR pathway that are directly related to disease progression [15, 39–42].

To utilize a new functional assay for clinical interpretation of a variant, it is necessary to test a sufficient number of known controls to calibrate the assay. Brnich et al. calculated that assays should test at least 11 known controls with a mix of B/LB and P/LP variants. Under such circumstances, an OddsPath_Functional score can be obtained, which can then be equated with a descriptive strength of evidence category as utilized by the ACMG/AMP variant interpretation guidelines [24]. The newly approved ClinGen/InSiGHT guidelines for MMR variants indicate that a functional assay that results in a variant OddsPath_Functional score > 18.7 can be used at the PS3 evidence level, while > 4.3 can be considered PS3_Moderate and > 2.08 as PS3_Supporting. Conversely, an OddsPath_Functional score < 0.05 for a variant could be used as BS3 evidence and < 0.48 as BS3_Supporting. In this study, we tested 12 known control variants for both *MSH6* and *MSH2*, including an equal number of B/LB and P/LP variants. As inCAMA encompasses two distinct functional assays, we tested the control calibration variants in each assay. By using a statistical clustering analysis, we showed that our B/LB and P/LP calibration variants for both *MSH6* and *MSH2* clustered into two distinct populations in both assays that were entirely concordant with normal and abnormal MMR function, respectively. Thus, the 12 variants we tested for each gene were sufficient to allow us to calculate OddsPath_Functional scores for each individual assay that can then be combined into a final score for a given variant.

To test the ability of this approach to calculate accurate OddsPath_Functional scores, we examined an additional set of known B/LB and P/LP variants with inCAMA. For each of these test variants, the calculated OddsPath_Functional scores provided strong evidence of benign or pathogenic status as expected, with one exception (Table 3). The Arg468His variant assay results led to an OddsPath_Functional score of 0.49, which just misses the threshold for BS3_Supporting evidence (Supporting Information 2: Tables S10 and S11). The variant is currently classified as LB even without functional data. One study describes a patient carrying this variant who has a microsatellite stable (MSS) tumor [43] while another study describes a patient with an MSI-low tumor and a lack of family history of LS-associated cancer [4]. Bioinformatic approaches show conflicting results, as SIFT predicts that this variant is tolerant while PolyPhen predicts it as possibly damaging [44]. The MAPP/PP2 score for this variant is 0.1095, which provides BP4_Supporting evidence. The inability to obtain BS3 evidence for Arg468His stems from the fact that this was the only variant to show an unexpected result in our MNNG survival assay, with relative loss of the DNA damage response despite its expected normal function. However, this variant showed normal activity in the MSI assay, which is consistent with the normal function displayed when tested previously by CIMRA [25]. CIMRA measures the ability of the mutated protein to repair a mismatch in an artificial DNA template and thus is another measure of the direct repair function. CIMRA is also the only other current functional assay that has been calibrated such that an OddsPath_Functional score for MMR variants can be generated.

Our discordant results for Arg468His may call into question the benefit of combining two quantitative OddsPath_Functional subscores with inCAMA, as it prevented us from obtaining BS3 level evidence for this variant. However, for all the other variants tested here, the use of dual assays only strengthened confidence in their final result. We believe that the more rigorous dual testing is preferable, though the ClinGen/InSiGHT Expert Panel may make a judgment call in these cases where such discordant results exist. While it is rare to see our two subassays display conflicting results, we have observed it on occasion [26, 27]. The discovery of these discordant results as determined by inCAMA is of increased value when considering data from multiplex assays of variant effect (MAVEs), which have been utilized previously for variants in *MSH2* [45]. While these high-throughput assays are very attractive for their ability to assess a large number of variants rapidly [46, 47], their readout is based on the effect of a variant on the MMR-dependent DNA damage response only and not their direct repair function, which may limit their accuracy for occasional variants. If we had only utilized the DNA damage response results in our study, we would have generated PS3 level evidence for Arg468His, which would have been inconsistent with its current LB status and the existing CIMRA results. This variant provides a cautionary example for the use of MAVE for MMR variants going forward. The mechanistic rationale for the conflicting MMR functional results for these few variants such as Arg468His is unclear, though the cell lines we have generated should provide valuable tools to study this question further.

Another limitation of currently existing functional assays including CIMRA [22] and MAVE approaches [45] is the creation of the variant in a cDNA context. While this can work well for examining the effect of a variant on the encoded protein product, the fact that inCAMA involves the generation of the variant in the endogenous gene inside the cell expands our ability to examine a variant's effect at the mRNA level as well. Thus, variants that can impact MMR gene expression or mRNA splicing should be revealed. While an exhaustive examination of the effects of variants on splicing was beyond the objectives of the current study, we did test one *MSH6* variant c.4001G>C (Arg1334Gln) due to the complete loss of MSH6 protein and significant loss of function in these cells. This c.4001G>C variant impacts the last nucleotide of Exon 9 and was shown in a recent study to be associated with the skipping of Exon 9 in cDNA derived from a patient blood sample [34]. Consistent with this prior clinical observation, we also saw complete skipping of Exon 9 in our Arg1334Gln cells, emphasizing the capacity of our model to recreate splicing effects of variants observed in patients. Importantly, this result emphasizes the ability of inCAMA to uniquely detect nonprotein impacts of variants that will be missed by other existing approaches. This further opens the possibility to interrogate variants in noncoding portions of the MMR genes including the promoter and introns.

In the current study, we focused on previously classified control variants to calibrate inCAMA. Using a second testing set of variants that were originally classified as B/LB or P/LP by the InSiGHT VIC demonstrated the effectiveness of inCAMA in providing complementary functional evidence in support of their status. However, with the release of the new ClinGen/InSiGHT MMR gene classification guidelines, a number of these variants were downgraded to VUS due to slightly more stringent criteria for certain evidence categories. As a result, the OddsPath_Functional scores we generated for these variants with inCAMA can now actually help to reclassify these VUS as LP or LB, while a couple of them should now have sufficient evidence to reach P or B status (Table 3). A similar approach can be taken in the future to examine additional *MSH6* or *MSH2* VUS and aid in their reclassification.

## Figures and Tables

**Table 1:** Microsatellite instability in *MSH6* variant-expressing cell lines.

**Variant/MSI marker**	**BAT-40 ** ^**a**^	**MONO-27 ** ^**a**^	**MONO-51 ** ^**a**^	
WT	0/32	0/32	0/32
MSH6 KO	31/32	24/32	24/32
Calibration variants			
Benign/likely benign			
A20V	3/32	3/30	0/32
S144I	2/32	0/31	0/32
L396V	0/31	0/31	0/32
Y556F	5/32	0/32	1/32
V878A	0/32	3/32	0/32
P1073S	1/32	0/32	0/32
Pathogenic/likely pathogenic			
H367R	22/32	21/32	30/32
L449P	27/32	27/32	23/32
V480E	31/31	21/31	30/32
T767I	27/32	21/31	21/32
R772W	31/32	24/30	30/32
L1063R	30/32	30/31	27/32
Testing variants				
Benign/likely benign				
E220D	2/28	1/32	0/32	
F340S	0/32	0/32	0/32	
R468H	0/28	5/31	3/32	
V509A	0/31	3/32	0/32	
V800L	0/32	2/31	0/31	
R922Q	1/32	2/32	0/32	
E1163V	2/31	3/32	0/32	
R1304K	1/32	0/29	0/32	
S1329L	2/31	1/31	0/32	
Pathogenic/likely pathogenic				
V398E	27/31	26/26	32/32	
R482P	29/32	27/32	32/32	
S541R	28/32	20/32	31/32	
G686D	27/32	22/31	27/32	
F706S	31/31	29/31	27/32	
A1055P	29/32	31/32	22/32	
D1058H	17/32	30/32	29/32	
R1076C	18/31	23/32	31/32	
L1211P	20/31	22/29	21/32	
R1334Q	21/32	31/32	14/32	

Abbreviations: KO, MSH6 knockout hESCs; MSI, microsatellite instability; WT, wild-type hESCs.

^a^Ratio of clones with altered alleles compared with WT sequence to total clones tested.

**Table 2:** Microsatellite instability in MSH2 variant-expressing cell lines.

**Variant/MSI marker**	**NR-27 ** ^**a**^	**BAT-25 ** ^**a**^	**BAT-26 ** ^**a**^
WT	0/32	0/32	0/32
MSH2 KO	18/32	32/32	32/32
Calibration variants			
Benign/likely benign			
T8M	0/32	0/32	0/32
R106K	0/32	0/32	0/31
D167H	0/32	0/32	2/32
A272V	0/32	0/31	0/31
G322D	0/31	0/31	0/32
L390F	0/30	0/31	0/32
Pathogenic/likely pathogenic			
C199R	15/32	26/32	32/32
P622L	20/32	21/32	26/32
A636P	29/32	17/32	29/32
G669V	30/32	27/32	30/32
C697R	27/32	31/32	27/32
G751R	31/32	22/32	29/32
Testing variants			
Benign/likely benign			
N127S	2/28	1/32	0/32
Q419K	0/32	0/32	0/32
T564A	0/32	0/30	0/32
Q629R	0/32	0/32	0/32
Pathogenic/likely pathogenic			
L187R	19/32	30/30	23/32
G338E	22/32	30/32	32/32
P349R	19/30	30/30	31/32
L440P	18/31	17/29	11/32
P696L	23/29	11/32	31/32

Abbreviations: KO, MSH2 knockout hESCs; MSI, microsatellite instability; WT, wild-type hESCs.

^a^Ratio of clones with altered alleles compared with WT sequence to total clones tested.

**Table 3:** Summary of functional assays and predicted classifications for testing set variants.

**Variant**	**DNA damage response ** ^**a**^	**DNA repair ** ^**a**^	**OddsPath_Functional score ** ^**b**^	**Current ClinGen/InSiGHT classification ** ^**c**^	**ACMG/AMP evidence code ** ^**d**^	**Predicted updated classification ** ^**e**^

MSH6 variants						
Benign/likely benign^f^						
E220D	+	+	1.85e−04	LB	**BS3**, BS1, BP4_Sup	B
F340S	+	+	1.56e−04	VUS	**BS3**, BP4_Sup	LB
R468H	—	+	0.49	LB	BP5_Sup, BP4_Sup	LB
V509A^g^	+	+	1.79e−04	VUS	**BS3**, PP3_Sup	VUS
V800L	+	+	2.03e−04	VUS	**BS3**, BP4_Sup	LB
R922Q	+	+	2.55e−04	VUS	**BS3**, BP4_Sup, PM2_Sup	LB
E1163V	+	+	2.15e−04	B	**BS3**, BA1	B
R1304K	+	+	2.43e−04	B	**BS3**, BA1, BP4_Sup	B
S1329L	+	+	2.13e−04	VUS	**BS3**, BP4_Sup	LB
Pathogenic/likely pathogenic^f^						
V398E	—	—	2.57e+04	VUS	**PS3**, PP4_Mod, PP3_Mod, PM2_Sup	LP
R482P	—	—	7590	VUS	**PS3**, PP4, PP3_Mod, PM2_Sup	LP
S541R	—	—	1.66e+04	VUS	**PS3**, PP1_Sup, PP3_Mod, PM2_Sup	LP
G686D	—	—	1.02e+04	VUS	**PS3**, PP4, PP1_Sup, PP3_Mod, PM2_Sup	LP
F706S	—	—	2.06e+04	VUS	**PS3**, PP4, PP3_Mod, PM2_Sup	LP
A1055P	—	—	3.24e+04	VUS	**PS3**, PP4, PP1_Sup, PP3_Sup, PM2_Sup	LP
D1058H	—	—	4070	VUS	**PS3**, PP4, PP1_Sup, PP3_Mod, PM2_Sup	LP
R1076C	—	—	120	LP	**PS3**, PP3_Sup, PM2_Sup, PM3_Mod	P
L1211P	—	—	1260	LP	**PS3**, PP4, PP1_Mod, PP3_Mod, PM2_Sup	P
R1334Q	—	—	7940	LP	**PS3**, PP4_Strong, PP1_Mod, BP4_Sup, PM2_Sup	P
MSH2 variants						
Benign/likely benign^f^						
N127S	+	+	9.25e−07	B	**BS3**, BA1	B
Q419K	+	+	2.05e−06	B	**BS3**, BP5_Sup, BP4_Sup, BA1	B
T564A	+	+	1.56e−06	LB	**BS3**, BP5_Strong, BP4_Sup	B
Q629R	+	+	2.77e−06	B	**BS3**, BP5_Sup, BS4_Strong, BP4_Sup	B
Pathogenic/likely pathogenic^f^						
L187R	—	—	1990	VUS	**PS3**, PP4, PP1_Sup, PP3_Mod, PM2_Sup	LP
G338E	—	—	8750	VUS	**PS3**, PP4_Mod, PP3_Mod, PM2_Sup	LP
P349R	—	—	7690	VUS	**PS3**, PP4_Mod, PP3_Mod, PM2_Sup	LP
L440P	—	+/−	91.8	VUS	**PS3**, PP4_Mod, PP3_Mod, PM2_Sup	LP

**Variant**	**DNA damage response**	**DNA repair**	**OddsPath_Functional score**	**Current ClinGen/InSiGHT classification**	**ACMG/AMP evidence code**	**Predicted updated classification**

P696L	—	—	589	VUS	**PS3**, PP4_Mod, PP3_Mod, PM2_Supp	LP

^a^From this study.

^b^OddsPath_Functional scores calculated as described in the Methods section.

^c^Current ClinGen/InSiGHT classifications in accordance with new MMR gene variant classification guidelines (https://cspec.genome.network).

^d^Evidence codes for all available data for each variant in accordance with new MMR gene variant classification guidelines. The evidence codes generated from the OddsPath_Functional scores derived in this study are in bold: **PS3**, strong pathogenic; **BS3**, strong benign.

^e^Predicted updated classification when combining OddsPath_Functional score from this study with other existing evidence codes.

^f^Classification headings based on original posterior probability of pathogenicity scores.

^g^Original classification due to minor allele frequency > 1% in a specific population.

## Data Availability

The data that support the findings of this study are available from the corresponding author upon reasonable request.

## Supporting Information

Supporting Information 1 Figure S1. The Sanger sequencing chromatogram of the targeted regions for each *MSH6* variant-expressing cell line created for this study is shown. The single nucleotide change is highlighted by a red star, and the cDNA change is given. Figure S2. Steady-state levels of MSH6 and MSH2 proteins. Representative Western blot images of MSH6 and MSH2 steady-state protein levels in cells expressing (A) benign/likely benign calibration variants, (B) pathogenic/likely pathogenic calibration variants, (C) benign/likely benign testing set variants, and (D) pathogenic/likely pathogenic testing set variants. Actin was probed as a loading control. The quantitation of MSH6 and MSH2 levels with respect to WT levels is shown after normalizing for loading. Figure S3. MNNG cell survival assay for multiple MSH6 R468H clones. Cell survival (percentage) for two independent R468H targeted clones after 48-h treatment with 1 *μ*M of MNNG, along with WT and *MSH6* knockout (KO) controls. The values are represented as the mean ± standard error of the mean; *n* = 7. Figure S4. Statistical clustering for the MSI results for *MSH6* variants. Graphs demonstrating percentage of unstable clones for WT, *MSH6* knockout (KO), and the B/LB (green) and P/LP (red) calibration control variants and testing set (gold) variants for the MSI markers BAT-40, MONO-27, and MONO-51. Statistical clustering was performed to segregate the results into two separate clusters (blue and orange circles). Figure S5. The Sanger sequencing chromatogram of the targeted regions for each *MSH2* variant-expressing cell line created for this study is shown. The single nucleotide change is highlighted by a red star, and the cDNA change is given. Figure S6. Statistical clustering for the MSI results for *MSH2* variants. Graphs demonstrating percentage of unstable clones for WT, *MSH2* knockout (KO), the B/LB (green) and P/LP (red) calibration control variants, and testing set (gold) variants for the MSI markers NR-27, BAT-25, and BAT-26. Statistical clustering was performed to segregate the results into two separate clusters (blue and orange circles).

Supporting Information 2 Table S1. Sequences of MSH6 variant sgRNAs and single strand oligonucleotides used for gene editing. Table S2. Sequences of MSH2 variant sgRNAs and single strand oligonucleotides used for gene editing. Table S3. Primers used to genotype *MSH6* variant carrying cell lines. Table S4. Primers used to genotype *MSH2* variant carrying cell lines. Table S5. Primers used to amplify microsatellite markers for MSI analyses. Table S6. Characteristics of *MSH6* variants examined in this study. Table S7. Characteristics of *MSH2* variants examined in this study. Table S8. Results of sequencing analyses of top five candidate loci for off-target cleavage by CRISPR nuclease for each *MSH6* variant cell line created in this study. Table S9. Results of sequencing analyses of top five candidate loci for off-target cleavage by CRISPR nuclease for each *MSH2* variant cell line created in this study. Table S10. Individual odds of pathogenicity scores for the MNNG survival and MSI assays performed for each *MSH6* testing variant. Table S11. Individual odds of pathogenicity scores for the MNNG survival and MSI assays performed for each *MSH2* testing variant.
