# Supplementary material for: A Cell-Based Functional Assay Calibrated for Analysis of MSH6 and MSH2 Mismatch Repair Gene Variants
Source: Hum Mutat. 2025 Sep 6;2025:3923193. doi: 10.1155/humu/3923193 (PMC12433325; doi:10.1155/humu/3923193)
Supplement: Supporting Information 1 — Figure S1. The Sanger sequencing chromatogram of the targeted regions for each MSH6 variant-expressing cell line created for this study is shown. The single nucleotide change is highlighted by a red star, and the cDNA change is given. Figure S2. Steady-state levels of MSH6 and MSH2 proteins. Representative Western blot images of MSH6 and MSH2 steady-state protein levels in cells expressing (A) benign/likely benign calibration variants, (B) pathogenic/likely pathogenic calibration variants, (C) benign/likely benign testing set variants, and (D) pathogenic/likely pathogenic testing set variants. Actin was probed as a loading control. The quantitation of MSH6 and MSH2 levels with respect to WT levels is shown after normalizing for loading. Figure S3. MNNG cell survival assay for multiple MSH6 R468H clones. Cell survival (percentage) for two independent R468H targeted clones after 48-h treatment with 1 μM of MNNG, along with WT and MSH6 knockout (KO) controls. The values are represented as the mean ± standard error of the mean; n = 7. Figure S4. Statistical clustering for the MSI results for MSH6 variants. Graphs demonstrating percentage of unstable clones for WT, MSH6 knockout (KO), and the B/LB (green) and P/LP (red) calibration control variants and testing set (gold) variants for the MSI markers BAT-40, MONO-27, and MONO-51. Statistical clustering was performed to segregate the results into two separate clusters (blue and orange circles). Figure S5. The Sanger sequencing chromatogram of the targeted regions for each MSH2 variant-expressing cell line created for this study is shown. The single nucleotide change is highlighted by a red star, and the cDNA change is given. Figure S6. Statistical clustering for the MSI results for MSH2 variants. Graphs demonstrating percentage of unstable clones for WT, MSH2 knockout (KO), the B/LB (green) and P/LP (red) calibration control variants, and testing set (gold) variants for the MSI markers NR-27, BAT-25, and BAT-26. Statistica [file 3923193.f1.pdf]

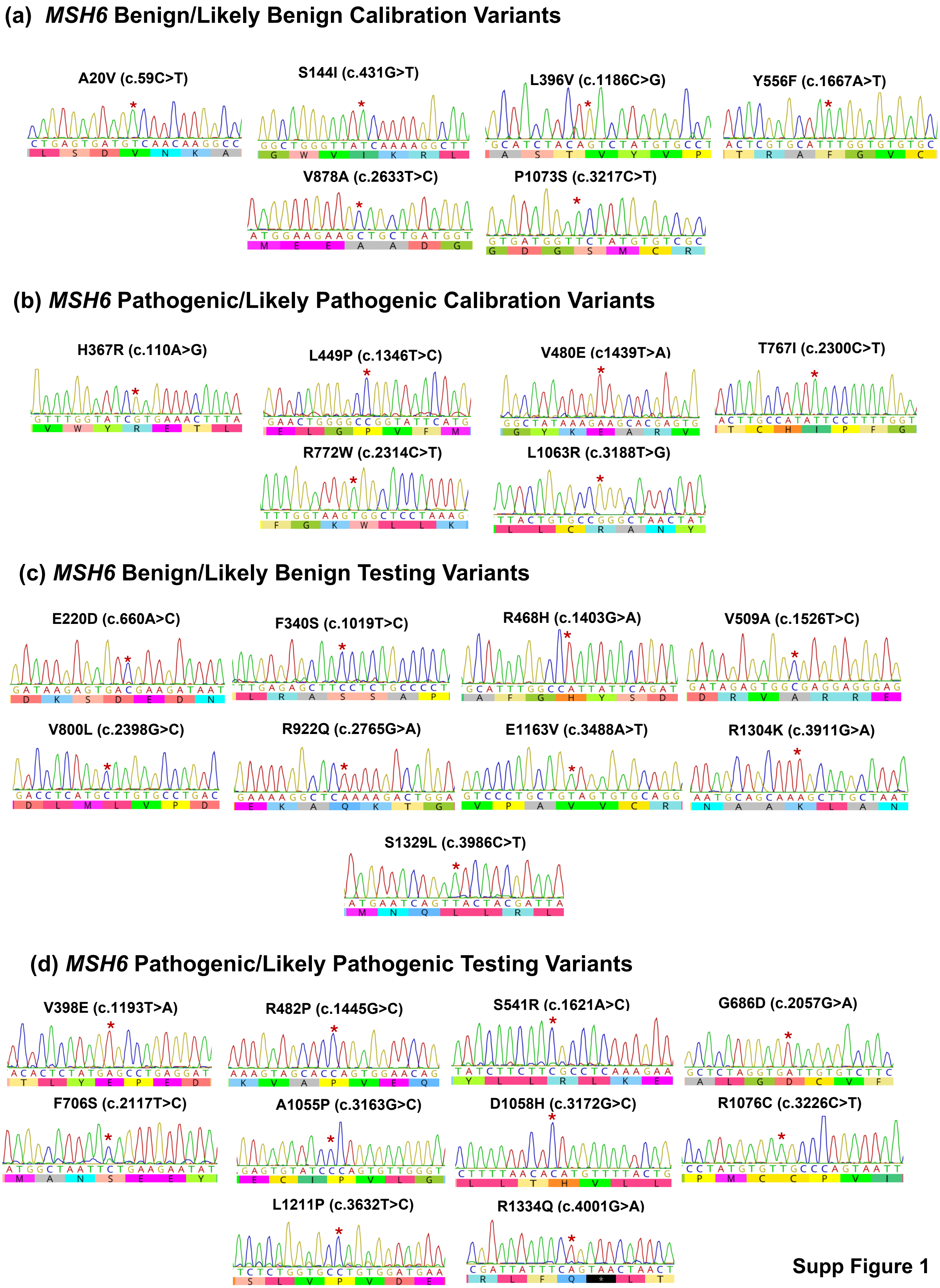

Supp Figure 1

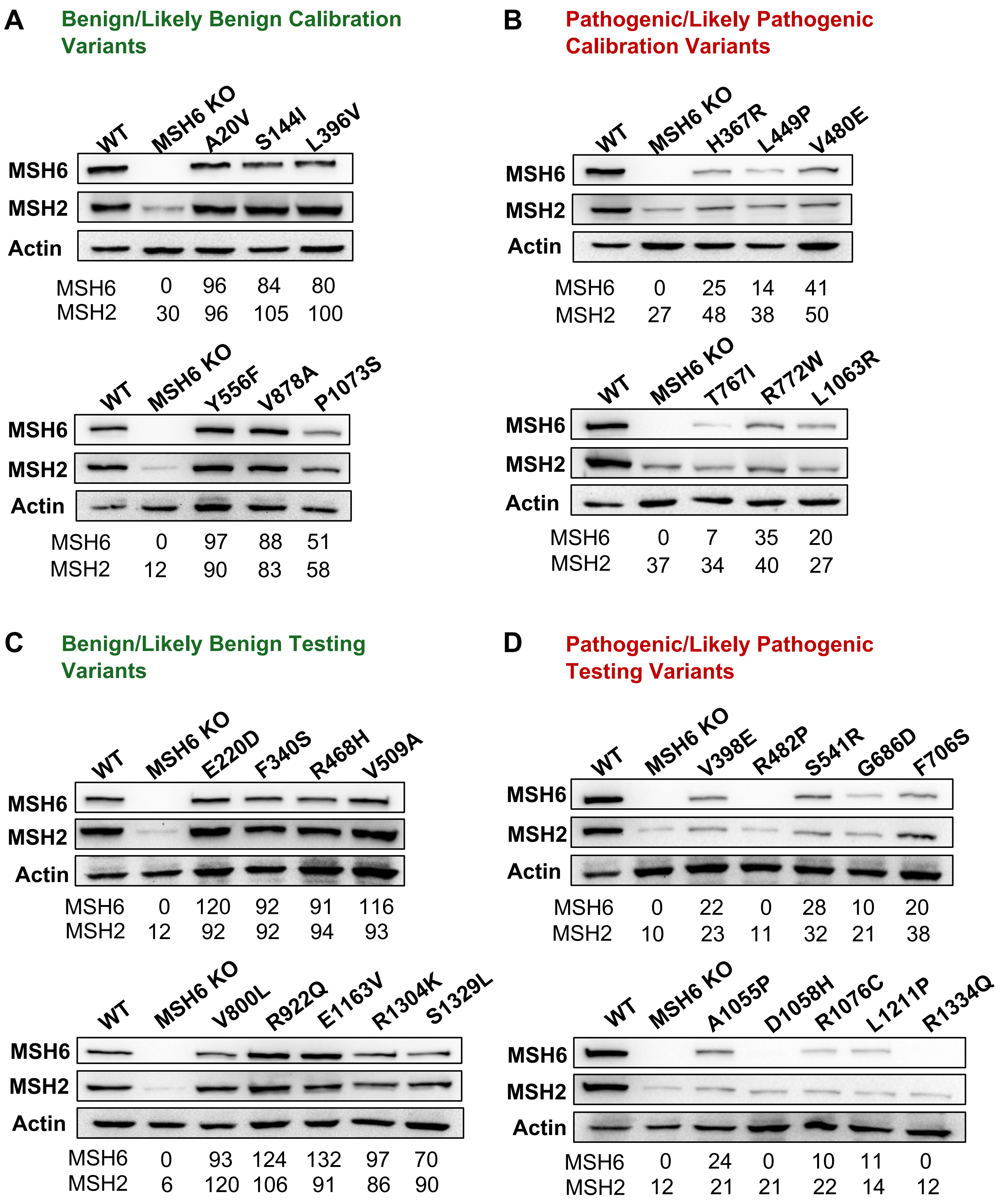

Supp Figure 2

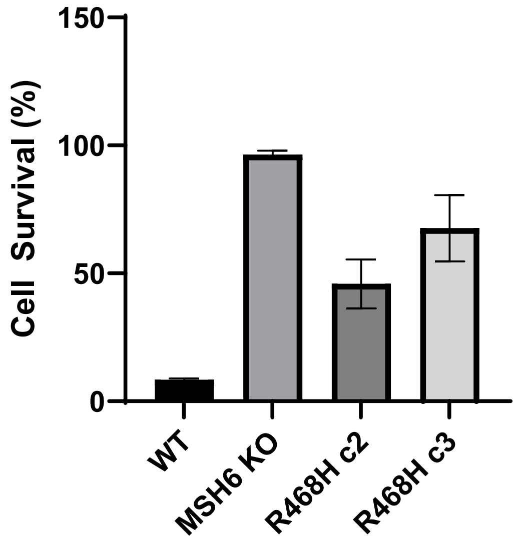

Supp Figure S3

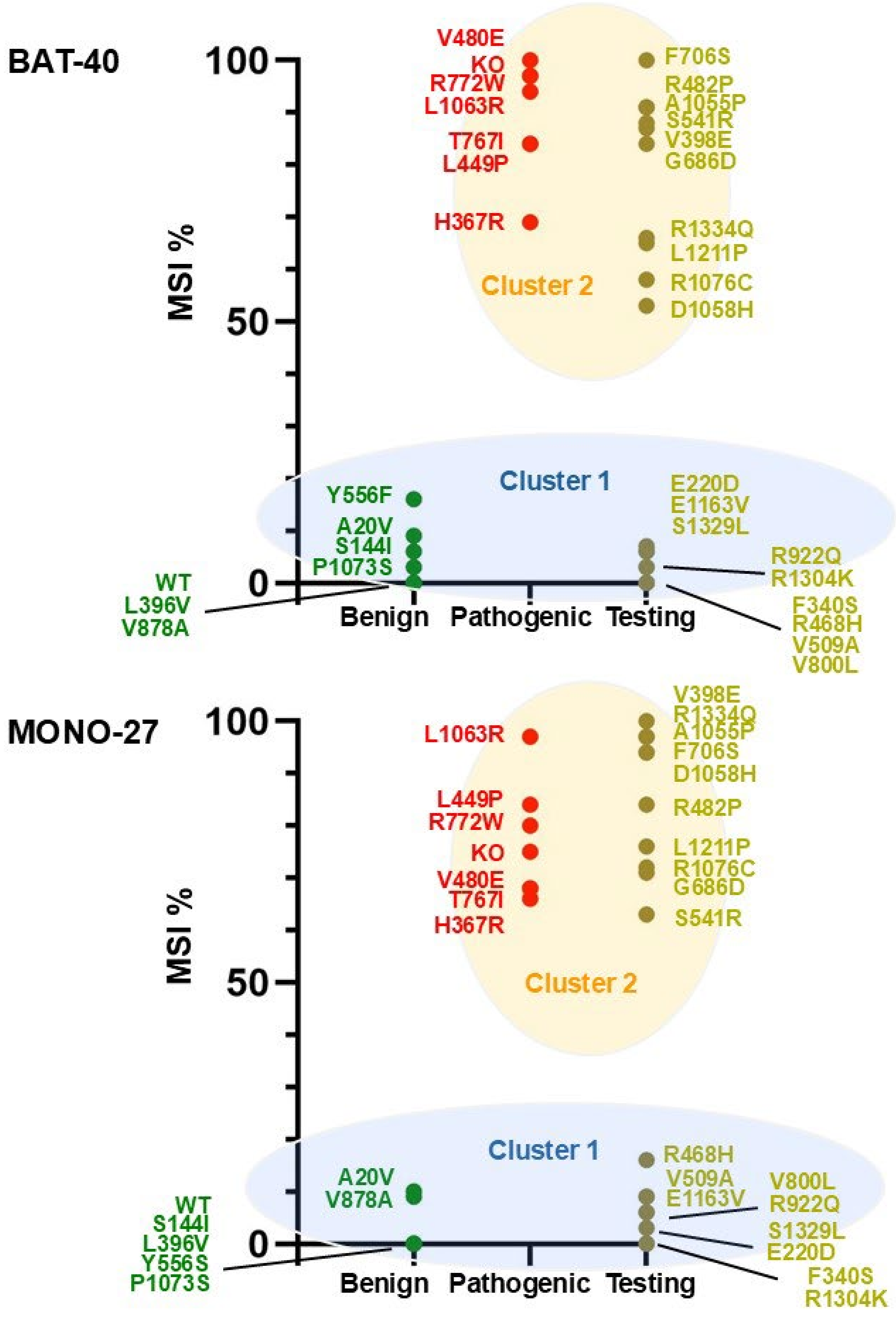

BAT-40
MSI %
100
50
0
V480E
KO
R772W
L1063R
T767I
L449P
H367R
F706S
R482P
A1055P
S541R
V398E
G686D
R1334Q
L1211P
R1076C
D1058H
Cluster 2
Cluster 1
Y556F
A20V
S144I
P1073S
WT
L396V
V878A
E220D
E1163V
S1329L
R922Q
R1304K
F340S
R468H
V509A
V800L
Benign
Pathogenic
Testing
MONO-27
MSI %
100
50
0
L1063R
L449P
R772W
KO
V480E
T767I
H367R
V398E
R1334Q
A1055P
F706S
D1058H
R482P
L1211P
R1076C
G686D
S541R
Cluster 2
Cluster 1
A20V
V878A
WT
S144I
L396V
Y556S
P1073S
R468H
V509A
E1163V
V800L
R922Q
S1329L
E220D
F340S
R1304K
Benign
Pathogenic
Testing

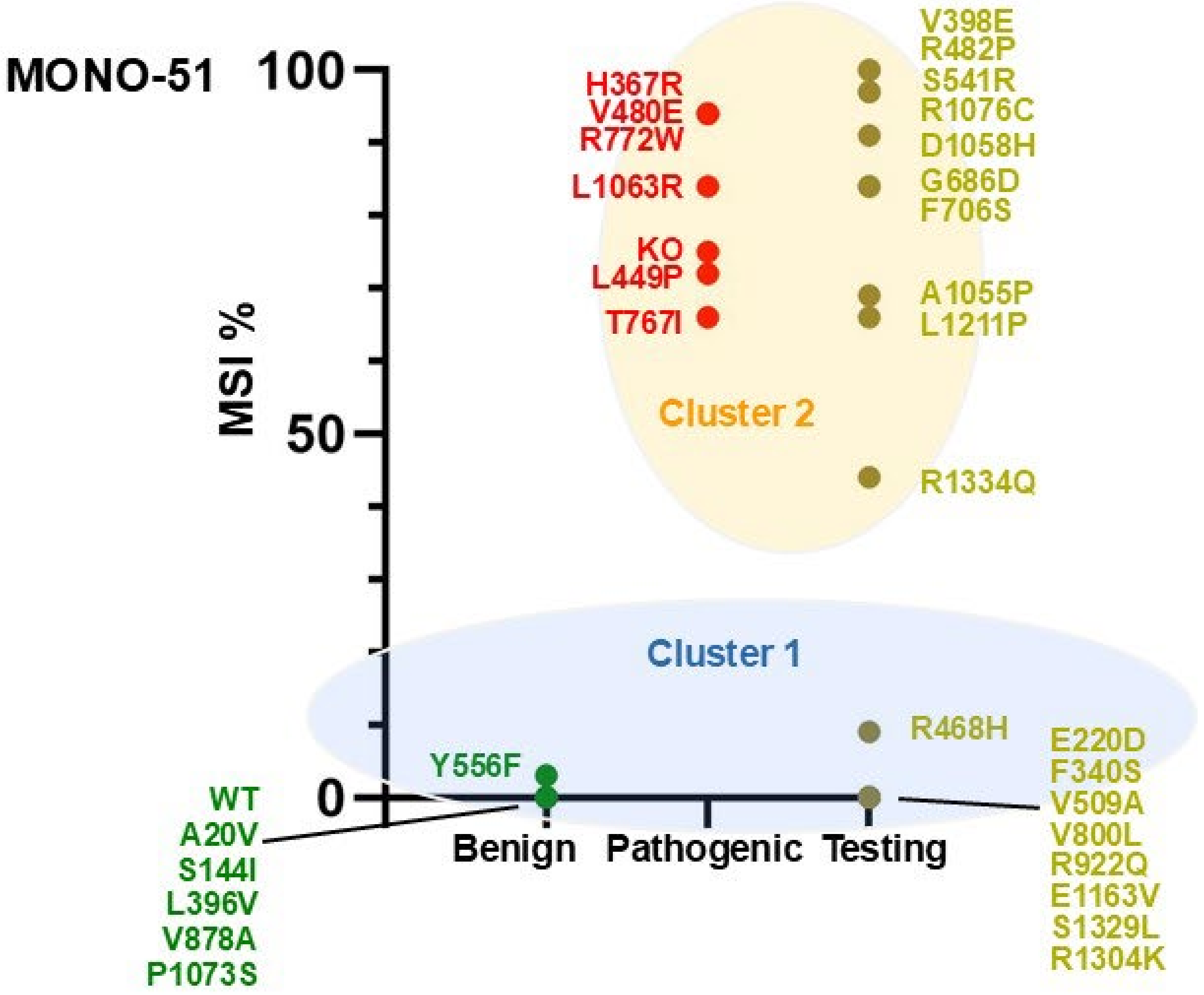

Supp Figure S4

(a) *MSH2* Benign/Likely Benign Calibration Variants

R106K (c.317G>A)

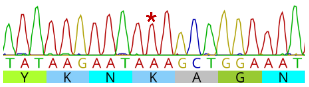

A272V (c.815C>T)

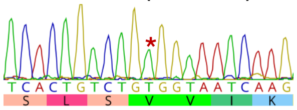

(b) *MSH2* Pathogenic/Likely Pathogenic Calibration Variants

A636P (c.1906G>C)

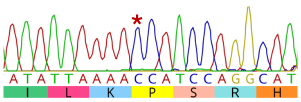

G669V (c.2006G>T)

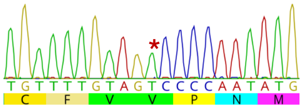

(c) *MSH2* Benign/Likely Benign Testing Variants

Q419K (c.1255C>A)

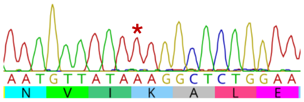

T564A (c.1690A>G)

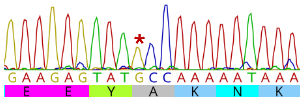

Q629R (c.1886A>G)

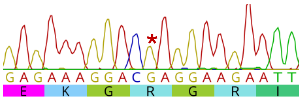

(d) *MSH2* Pathogenic/Likely Pathogenic Testing Variants

L187R (c.560T>G)

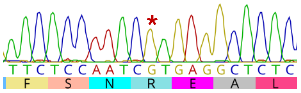

G338E (c.1013G>A)

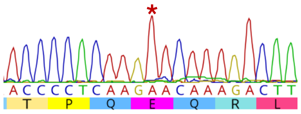

L440P (c.1319T>C)

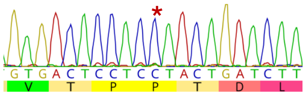

P696L (c.2087C>T)

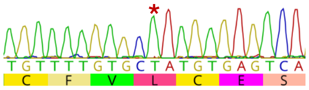

Supp Figure S5

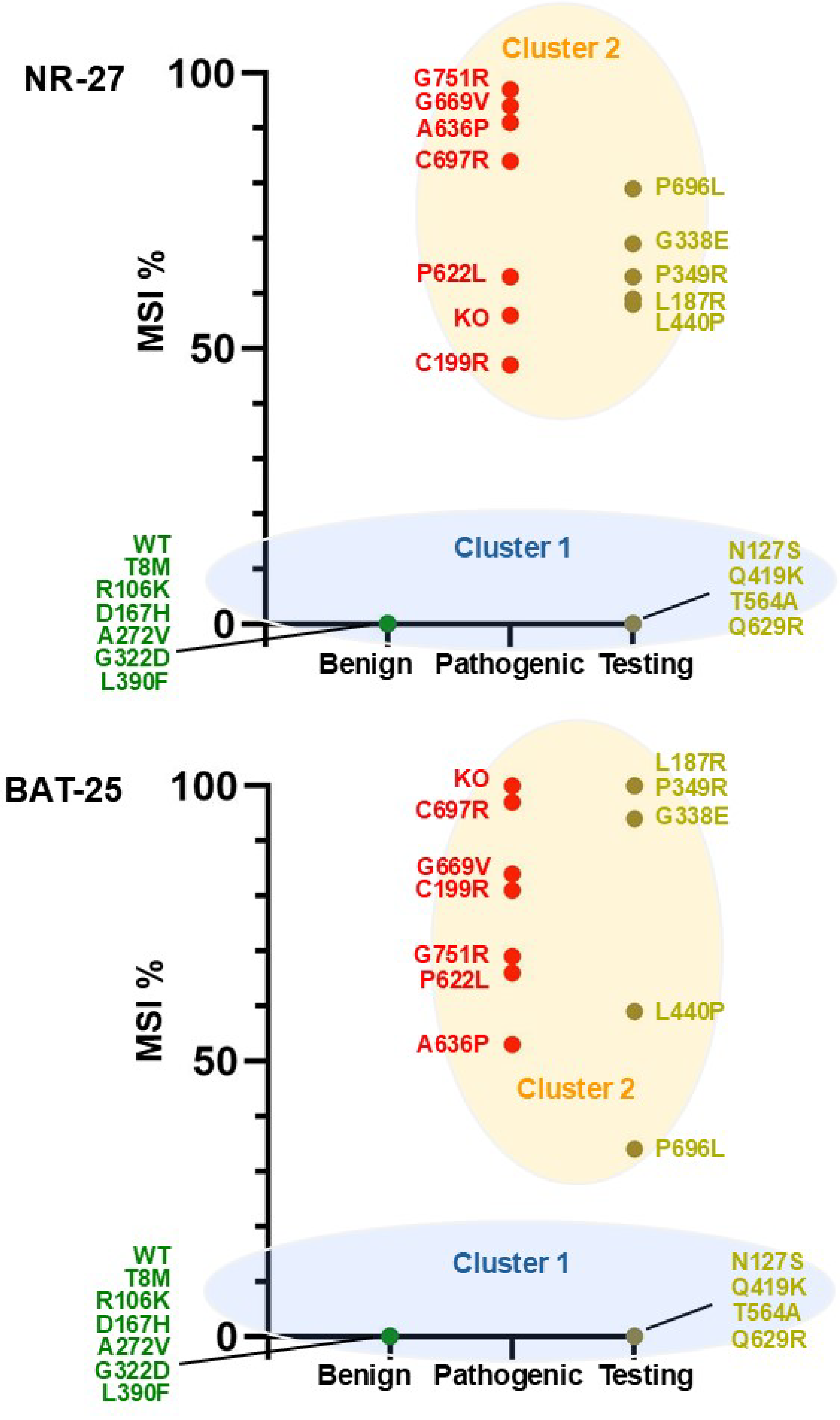

NR-27
Cluster 2
100
50
0
MSI %
G751R
G669V
A636P
C697R
P622L
KO
C199R
P696L
G338E
P349R
L187R
L440P
Cluster 1
WT
T8M
R106K
D167H
A272V
G322D
L390F
N127S
Q419K
T564A
Q629R
Benign
Pathogenic
Testing
BAT-25
100
50
0
MSI %
KO
C697R
G669V
C199R
G751R
P622L
A636P
L187R
P349R
G338E
L440P
Cluster 2
P696L
Cluster 1
WT
T8M
R106K
D167H
A272V
G322D
L390F
N127S
Q419K
T564A
Q629R
Benign
Pathogenic
Testing

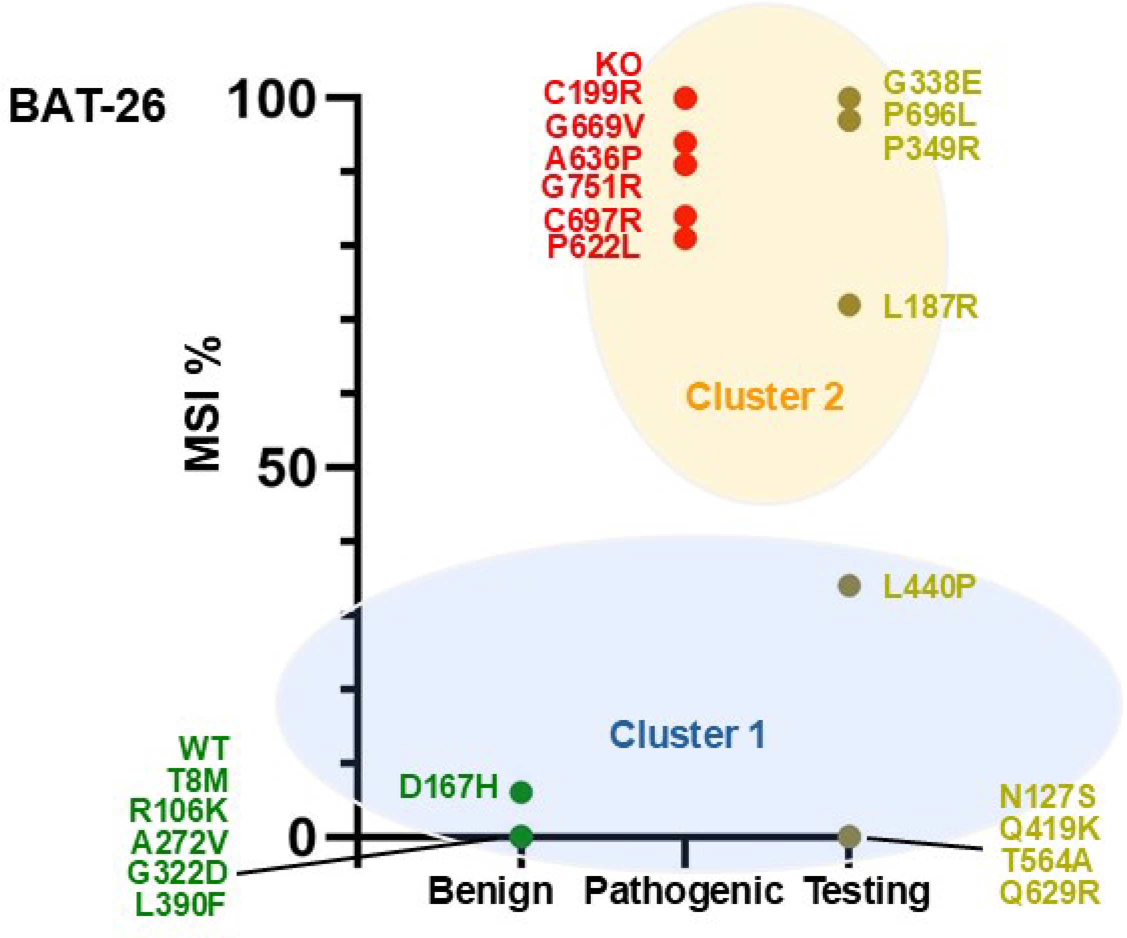

Supp Figure S6

TABLE S3 Primers used to genotype the *MSH6* variant carrying cell lines

Cell Line	Forward Primer	Reverse Primer
MSH6 KO	AGGAAGTGGTGGCAGATTAAG	CACGAGTATGGCCAGAAGAAT
	**Calibration Variants**	
Benign/Likely Benign		
A20V	CGGTAGATGCGGTGCTTT	TTGAGGTTCTTCGCCTTGG
S144I	TGACTTCTCACCAGGAGATTTG	GTTGTGAAAGCTTCTGGCATG
L396V	CGCCCTACTGTTTGGTATCAT	AAGAGCATCCATGTGGTACAG
Y556F	GATGGAGGCACGATGTAGAA	CCTGAAGAGAACAGGACAATGA
V878A	TCTTTCTGCTCTGGAAGGATTC	CCAGGAGGCTCTGTTCATTT
P1073S	GAGGGTAAGTATTTTGATGGG	CAAGCACACAATAGGCTTTG
Pathogenic/Likely Pathogenic		
H367R	TCGAAAGCGGAAGAGAATGG	GAGGAAATCCTCAGGCACATAG
L449P	CTACACTCTATGTGCCTGAG	CTTGGTAATGATCCTACAG
V480E	CTTGTCATCTGTTACAAGG	CTCTTTTTCTTTGAGGCTAAG
T767I	GCCTTATTGATCAGGAGC	CTCAAGATCTGGAAGCTTC
R772W	GACACAGTCAGCACTACAAGAT	TTGTCAGGCACAACCATGA
L1063R	GCTCTATCTTTTAGCTTTCC	CTCACAGCCTATTAGAATGTC
	**Testing Variants**	
Benign/Likely Benign		
E220D	CTATCTCAGACGTGCTCAG	AATGTCACTCTCAGAATCTGA
F340S	GAGGAAGGAAGCAGTGATGAA	GAGGAAATCCTCAGGCACATAG
R468H	CATCTACACTCTATGTGCCTG	GATCCTACAGATCTCCCTCC
V509A	GGATGCTCTTATTGGAGTCAG	CACCATATGCACGAGTATGG
V800L	CTAGAGAGGGTTGATACTTGC	AAAACCATCAGCAACTTCTTC
R922Q	CTTTCTGCTCTGGAAGGATTCA	TGCGCTGTTTCTCTAGGTATTC
E1163V	GAGGCCCTATGCCTCTTG	CATTATTCTGTCTGAGGCACC
R1304K	CACTTCTCTTGCTAGCACATG	CATCCCTTCCCCTTTTACTG
S1329L	CCCAGCCAGGAGACTATTAC	CTTTCACTAGCCAGGCAAAC
Pathogenic/Likely Pathogenic		
V398E	GTCGCCCTACTGTTTGGTATC	CTGCACCAGGGAATCTGAATAA
R482P	GTACTCCTGGGATGAGGAAGT	GATCACCTTCCAGCACACTG
S541R	GGAGGCACGATGTAGAAAG	GGGGATAGTGTGCCACTAG
G686D	GAGAACTCTCCTTGAGGAAG	GTTCCATTCAGAAAAATCTC
F706S	CCATTGGGTTGACACCAG	GGCAAGTATCAACCCTCTC
A1055P	GTAAACGATACTGGACCAAAA	CCTGACTAGGCTGCCCACAG
D1058H	GGGTAAGTATTTTGATGGGGG	CTCACAGCCTATTAGAATGTC
R1076C	GATCGTTGGACTGTAATTG	CAGTAACAAGCACACAATAG
L1211P	TTGTTGAATTAAGTGAAACTGC	CAACTATCGGTCTGTGCC
R1334Q	TTTGAGAGGGCACTTCTCTTG	ATGGACAGCTTCAGCATCTAC

TABLE S4 Primers used to genotype the *MSH2* variant carrying cell lines

Cell Line	Forward Primer	Reverse Primer
	**Calibration Variants**	
Benign		
R106K	GAAGTCCAGCTAATACAGTGC	GACCAGCCAAACTGCAAC
A272V	GAGGGACTTCAGAATTTATT	ACTGCTGCAATATCCAATTTC
Pathogenic		
A636P	GCTAGATGCTGTTGTCAGCT	TTACCAGTAATGATGTGGAAC
G669V	CAGTAACTCTGTCCACATCTTTGG	TATATAAAGTCCACAGGAAAACAACTATTAA
	**Testing Variants**	
Benign/Likely Benign		
Q419K	CGATTCCCAGATCTTAACCGAC	TTTATGAGGACAGCACATTGCC
T564A	CCCTAAGGAGTTGTTCGTTTTC	CCTACTGTAACATACCATGCCT
Q629R	CAGGCTATGTAGAACCAATGCA	CGTTACCCCCACAAAGCC
Pathogenic		
L187R	GGCAATCTCTCTCAGTTTGAAG	GCCTGGAATCTCCTCTATCAC
G338E	CACTAATGAGCTTGCCATT	GTAACTGCAGGTTACATAAAAC
L440P	GGAGACCTGCTGTACTATTTG	CCACAAAGGTGCTACAATTAG
P696L	GGCTGTGGTTCTGCCTTTATATG	CAAGGGACTAGGAGATGCAC

TABLE S5 Microsatellite Marker Primers

MSI Marker	Forward Primer	Reverse Primer
BAT-40	6-FAM- CCTCAAGCCAAGATTAACTTCC	TGGTAGAGCAAGACCACCTT
MONO-27	6-FAM-CAGGGAAATGGTGGGAACCCA	AGTTCATGATGTGGTTCTGTC
MONO-51	6-FAM-TGGAATAATTGTGGCTTGCC	CTGGGAGAGTTCAATCACG
BAT-25	HEX-CTTTCCTCGCCTCCAAGAATG	CCACACTTCAAAATGACATTCTGC
BAT-26	6-FAM-CTGCGGTAATCAAGTTTT	GAACCATTCAACATTTTTAACCC
NR-27	6-FAM-AACCATGCTTGCAAACCACT	GCGATAATACTAGCAATGACC

TABLE S6 *MSH6* variant characteristics

Calibration Variants

Cell Line	cDNA Change	ClinVar ID	Oddspath_ Non-functional[a]
Benign/Likely Benign			
A20V	c.59C>T	89540	0.01
S144I	c.431G>T	41596	1.02E-05
L396V	c.1186C>G	36582	5.78E-06
Y556F	c.1667A>T	89214	0.03
V878A	c.2633T>C	8931	2.08E-06
P1073S	c.3217C>T	41593	0.01
Pathogenic/Likely Pathogenic			
H367R	c.1100A>G	520541	397
L449P	c.1346T>C	89189	8.62E06
V480E	c.1439T>A	633495	7.75E06
T767I	c.2300C>T	141058	184
R772W	c.2314C>T	89267	64.5
L1063R	c.3188T>G	410436	458

Testing Variants

Cell Line	cDNA Change	ClinVar ID	Oddspath_ Non-functional[a]
Benign/Likely Benign			
E220D	c.660A>C	89551	-
F340S	c.1019T>C	89165	0.01
R468H	c.1403G>A	89192	1.23E-03
V509A	c.1526T>C	41588	-
V800L	c.2398G>C	89279	0.01
R922Q	c.2765G>A	410514	0.01
E1163V	c.3488A>T	89400	0.37
R1304K	c.3911G>A	89479	-
S1329L	c.3986C>T	89497	0.01
Pathogenic/Likely Pathogenic			
V398E	c.1193T>A	89179	89.4
R482P	c.1445G>C	561174	36.5
S541R	c.1621A>C	126891	26.4
G686D	c.2057G>A	89245	81.6
F706S	c.2117T>C	89254	34.6
A1055P	c.3163G>C	126892	27.8
D1058H	c.3172G>C	218057	60.8
R1076C	c.3226C>T	89357	2.24
L1211P	c.3632T>C	219294	246
R1334Q	c.4001G>A	89506	103

[a]Odds of Pathogenicity calculated based on known tumor and family segregation data from patients harboring variant. Any prior functional data was excluded in the calculation. Input data for each variant can be found at http://insight-database.org/classifications/mmr_integrative_eval.html.

TABLE S7 *MSH2* variant characteristics

Calibration Variants

Cell Line	cDNA Change	ClinVar ID	Oddspath_ Non-functional
Benign			
T8M	c.23C>T	90964	1.22E-05
R106K	c.317G>A	91062	6.67E-04
D167H	c.499G>C	91112	2.14E-05
A272V	c.815C>T	41651	1.48E-05
G322D	c.965G>A	1762	4.60E-13
L390F	c.1168C>T	41641	1.43E-08
Pathogenic			
C199R	c.595T>C	91146	605
P622L	c.1865C>T	1753	99
A636P	c.1906G>C	1764	1.11E11
G669V	c.20006G>T	90854	286
C697R	c.2089T>C	90882	451
G751R	c.2251G>A	90943	230

Testing Variants

Cell Line	cDNA Change	ClinVar ID	Oddspath_ Non-functional
Benign/Likely Benign			
N127S	c.380A>G	36577	-
Q419K	c.1255C>A	90583	4.44E-03
T564A	c.1690A>G	90750	1.11E-04
Q629R	c.1886A>G	90812	6.67E-05
Pathogenic			
L187R	c.560T>G	91135	128
G338E	c.1013G>A	90504	79.2
P349R	c.1046C>G	90513	184
L440P	c.1319T>C	90625	111
P696L	c.2087C>T	90881	111

TABLE S8 Examination of top five candidate loci for off-target cleavage by CRISPR nuclease for *MSH6*

Cell Line	I	II	III	IV	V
MSH6 KO	✓	✓	✓	✓	✓
**Calibration Variants**					
Benign Likely/Benign					
A20V	✓	✓	✓	✓	✓
S144I	✓	✓	✓	✓	✓
L396V	✓	✓	✓	✓	✓
Y556F	✓	✓	✓	✓	✓
V878A	✓	✓	✓	✓	✓
P1073	✓	✓	✓	✓	✓
Pathogenic/Likely Pathogenic					
H367R	✓	✓	✓	✓	✓
L449P	✓	✓	✓	✓	✓
V480E	✓	✓	✓	✓	✓
T767I	✓	✓	✓	✓	✓
R772W	✓	✓	✓	✓	✓
L1063R	✓	✓	✓	✓	✓
**Testing Variants**					
Benign/Likely Benign					
E220D	✓	✓	✓	✓	✓
F340S	✓	✓	✓	✓	✓
R468H	✓	✓	✓	✓	✓
V509A	✓	✓	✓	✓	✓
V800L	✓	✓	✓	✓	✓
R922Q	✓	✓	✓	✓	✓
E1163V	✓	✓	✓	✓	✓
R1304K	✓	✓	✓	✓	✓
S1329L	✓	✓	✓	✓	✓
Pathogenic/Likely Pathogenic					
V398E	✓	✓	✓	✓	✓
R482P	✓	✓	✓	✓	✓
S541R	✓	✓	✓	✓	✓
G686D sgRNA1	✓	✓	✓	✓	✓
G686D sgRNA2	✓	✓	✓	✓	✓
F706S	✓	✓	✓	✓	✓
A1055P	✓	✓	✓	✓	✓
D1058H	✓	✓	✓	✓	✓
R1076C	✓	✓	✓	✓	✓
L1211P	✓	✓	✓	✓	✓
R1334Q	✓	✓	✓	✓	✓

✓ - Sequence at candidate off-target site matches expected WT sequence

TABLE S9 Examination of top five candidate loci for off-target cleavage by CRISPR nuclease for *MSH2*

	Calibration Variants				
**Cell Line**	**I**	**II**	**III**	**IV**	**V**
Benign					
R106K	✓	✓	✓	✓	✓
A272V	✓	✓	✓	✓	✓
Pathogenic					
A636P	✓	✓	✓	✓	✓
G669V	✓	✓	✓	✓	✓
	**Testing Variants**				
Benign/Likely Benign					
Q419K	✓	✓	✓	✓	✓
T564A	✓	✓	✓	✓	✓
Q629R	✓	✓	✓	✓	✓
Pathogenic					
L187R	✓	✓	✓	✓	✓
G338E	✓	✓	✓	✓	✓
L440P	✓	✓	✓	✓	✓
P696L	✓	✓	✓	✓	✓

✓ - Sequence at candidate off-target site matches expected WT sequence

TABLE S10 OddsPath_Functional scores for individual assays for *MSH6* testing variants

Variant	MNNG Survival Assay	MSI Assay	Combined
Benign/Likely Benign			
E220D	7.50E-05 ± 0.775	3.99E-04 ± 0662	1.85E-04
F340S	1.15E-04 ± 0.756	2.01E-04 ± 0.689	1.56E-04
R468H	209 ± 0.551	1.11E-03 ± 0.624	0.49
V509A	7.38E-05 ± 0.775	3.73E-4 ± 0.664	1.79E-04
V800L	1.30E-04 ± 0.751	3.03E-04 ± 0.672	2.03E-04
R922Q	1.65E-04 ± 0.741	3.73E-04 ± 0.664	2.55E-04
E1163V	9.45E-05 ± 0.765	5.62E-04 ± 0.648	2.15E-04
R1304K	2.38E-04 ± 0.725	2.47E-04 ± 0.681	2.43E-04
S1329L	1.13E-04 ± 0.757	3.73E-04± 0.664	2.13E-04
Pathogenic/Likely Pathogenic			
V398E	1.34E+-4 ± 0.688	6.97E+04 ± 1.06	2.57E+04
R482P	3150 ± 0.633	3.06E+04 ± 1.01	7590
S541R	4.51E+04 ± 0.737	4820 ± 0.908	1.66E+04
G686D	3.03E+04 ± 0.720	2600 ± 0.875	1.02E+04
F706S	1.38E+04 ± 0.689	3.76E+04 ± 1.02	2.06E+04
A1055P	9.31E+04 ± 0.768	8930 ± 0.942	3.24E+04
D1058H	6030 ± 0.657	2430 ± 0.871	4070
R1076C	19.3 ± 0.506	1140 ± 0.832	120
L1211P	4290 ± 0.645	290 ± 0.763	1260
R1334Q	2.34E+05 ± 0.809	290 ± 0.763	7940

TABLE S11 OddsPath_Functional scores for individual assays for *MSH2* testing variants

Variant	MNNG Survival Assay	MSI Assay	Combined
Benign/Likely Benign			
N127S	7.62E-07 ± 1.18	1.10E-06 ± 1.23	9.25E-07
Q419K	1.69E-06 ± 1.14	1.10E-06 ± 1.23	2.05E-06
T564A	2.09E-06 ± 1.13	1.10E-06 ± 1.23	1.56E-06
Q629R	6.29E-06 ± 1.09	1.10E-06 ± 1.23	2.77E-06
Pathogenic			
L187R	4310 ± 1.30	967 ± 1.23	1990
G338E	4810 ± 1.31	1.68E+04 ± 1.39	8750
P349R	4740 ± 1.31	1.28E+04 ± 1.37	7690
L440P	1.37E+04 ± 1.37	0.772 ± 0.973	91.8
P696L	2830 ± 1.28	149 ± 1.15	589
